# The protective impact of adapted trimebutine maleate-loaded nanostructured lipid carriers for alleviating the severity of acute colitis

**DOI:** 10.1080/10717544.2022.2050847

**Published:** 2022-03-17

**Authors:** Amira Motawea, Walaa Ebrahim Abd El Hady, Ghada Ahmed El-Emam

**Affiliations:** Department of Pharmaceutics, Faculty of Pharmacy, Mansoura University, Mansoura, Egypt

**Keywords:** Nanostructured lipid carriers (NLC), trimebutine maleate, ulcerative colitis, histopathological, oral delivery

## Abstract

Nanoparticles for colon-drug delivery were designed and evaluated to solve many discrepancy issues such as high adverse effects of released drugs, insufficient drug amount at diseased areas, and unintentionally premature drug release to noninflamed GIT regions. Herein, the goal of this work was to convert trimebutine maleate (TMB) into nanostructured lipid carriers (NLC) in order to improve its protective effects in ulcerative colitis. NLC of TMB was prepared by the hot homogenization followed by ultra-sonication method. A full 4^2^-factorial design was used to estimate the produced TMB-NLC. The study design included the exploration of the impact of two independent variables namely lipid mix amount and ratio (glyceryl mono stearate and capryol 90), surfactant concentration (0.5, 1, 1.5, and 2%), on the particle size, polydispersity index, and the entrapment efficiency (EE%). The protective activity of F9 was examined through macroscopical scores, histopathological changes, immunohistochemical localization of tumor necrosis factor-α (TNF-α) and examination of oxidative stress such as reduced glutathione (GSH), superoxide dismutase (SOD), and malondialdehyde (MDA) against acetic acid-induced colitis in rats. Consistent with our expectations, the orally administered optimized formula (F9) alleviated the severity of colitis in acetic acid-induced rat model of colitis likely owing to the controlled release compared to free TMB. We aimed to develop TMB-loaded NLC for the treatment of acute colitis with the goal of providing a superior drug safety profile over long-term remission and maintenance therapy.

## Introduction

Ulcerative colitis (UC) is an immune-mediated disease referred to as inflammatory bowel diseases (IBDs) with a multifactorial pathogenesis impacted by the environment, genetics, and the gut microbiota's composition (Wangchuk et al., 2015). Rectal hemorrhage, diarrhea, abdominal discomfort, vomiting, nausea, anorexia, and fever are all major complications of UC, which affects predominantly the mucosal lining of the colon and rectum (Anwer et al., 2020). The mucosal immune system has been identified as the primary mediator of intestinal inflammation and injury, with cytokines playing a key role in the initiation of inflammation. Proinflammatory cytokines such as tumor necrosis factor-α (TNF-α) and interleukin-1β (IL-1β), as well as prostaglandins (PGs) and leukotrienes (LTs), are produced when the intestinal immune system is activated (Abdel-Daim et al., 2015).

One of the validated approaches for inducing an experimental model of IBD is the intrarectal administration of acetic acid. Due to its resemblance to human IBD, this approach is often utilized as a simple model for screening prospective drugs (El-Azab et al., 2016).

Destruction of the mucosal barrier, ulceration, weight loss, hemorrhage, increased vasopermeability, and overproduction of proinflammatory cytokines are major contributing factors in the induction of colitis (Owusu et al., 2020).

Acetic acid induces colitis through the protonated form of the acid, which liberates protons within the intracellular space, causing severe intracellular acidification and epithelial destruction. Also, the cyclooxygenase and lipoxygenase pathways are activated in the inflammatory response triggered by acetic acid (Kondamudi et al., 2014).

Trimebutine maleate (TMB) (2-dimethylamino-2-phenyl butyl 3,4,5-trimethoxybenzoate hydrogen maleate) is categorized as the only enkephalin receptor agonist that expedites gastric emptying via inducing the evoke of several GIT agents like gastrin, glucagon, and motilin (Cho et al., 2010; Boziki et al., 2021). Likewise, TMB has proven efficacy for the treatment of functional bowel disorders, including dyspepsia, IBS, and postoperative ileus (Long et al., 2010). Unlike calcium channel blockers, TMB has a more intricate function on both calcium and potassium channels, which can enhance the symptoms proficiently via its dual modulating impacts (Long et al., 2010). Additionally, its weak agonist capacity for activation of intestinal opioid receptors; local anesthetic activity may be owing to blocking sodium currents in sensory neurons, causing relieve from abdominal pain and alteration of bowel habits (Chevalier et al., 2004; Chao et al., 2016). Nevertheless, the lack of drug specificity during UC therapy is the major drawback associated with current formulations.

Colon drug delivery systems (CDDSs) are an example of drug targeting systems that have promising developments in the area of local and systemic treatment. Simultaneously, targeting drugs to the colon is extremely valuable for local treatment of a variety of diseases such as UC, Cohn's disease, and colon cancer (Qelliny et al., 2019). Traditional dosage forms have a number of drawbacks, including substantial first-pass metabolism, side effects due to drug absorption in the upper gastrointestinal tract (GIT), and only limited amounts of the drug reaching inflammatory colon regions with lower therapeutic efficiency (Rathbone, 2011).

Nanosized drug delivery systems are also associated with reduced toxicity and sustained release profiles (Khosa et al., 2018). To begin, solid lipid nanoparticles (SLN) are formed by replacing the liquid lipid (oil) component of an oil/water emulsion with solid lipids; however, drug ejection and poor drug loading capacity were demonstrated to be issues with SLN; to address these limitations, the nanostructured lipid carrier (NLC) was developed as the second generation (Patil et al., 2016). They are created by well-ordered mixing of blends of solid lipids with liquid lipids, forming a distinctive nanostructure having a solid matrix at room temperature with improved properties (Gokce et al., 2012; El-Helw & Fahmy, 2015).

Nanostructured lipid carriers have many advantages, such as improving drug loading and minimizing the frequency of drug discharge during storage. This benefit may be realized due to their both highly disordered lipid structures, as well as their imperfect lipid matrix, which provides good housing for the drug molecules (How et al., 2011; Andalib et al., 2012). Among the other features of NLC are their controlled release profiles, and site-specific drug delivery, biodegradability of their lipid components, nontoxicity of breakdown products, a high capacity for incorporating both lipophilic and hydrophilic medicines, and the potential to improve the chemical stability of medications susceptible to light, oxidation, and hydrolysis (Rahman et al., 2013; Beloqui et al., 2016). In contrast to SLN, NLC has an inferior melting point regarding its oil content, while preserving its particulate nature of being solid at body temperature (Sanad et al., 2010).

The development and optimization of a new oral delivery system for TMB based on NLC, organized by the hot homogenization technique followed by ultrasonication, was the objective of the current study. The overall process was modulated using factorial design. First, it was applied to NLC formulation, wherein an examination of particle size, distribution homogeneity, and also entrapment efficiency percentage (EE%) was piloted. This experimental design means endorsing the immediate study of the impact of each independent variable upon the system and the corresponding interaction, using a restricted number of investigations (Vitorino et al., 2011).

Drug delivery in the form of NLC to the inflamed bowel has the ability to protect drugs from the GIT's environment and first-pass metabolism. NLC can also passively target inflamed areas, enhance drug deposition at the diseased site, prolong the desired pharmacological effect, and reduce the side effects of the drugs used in UC treatment (Ashkar et al., 2022).

Paracellular pathways or endocytosis has been extensively explored for the delivery of nanocarriers into intestinal epithelial cells. When the intestinal tissue becomes inflamed, the absorption of the nanobio-systems is enabled by the loss of the gastrointestinal mucus gel and the reduced intestinal epithelial barrier function (Nedelcu et al., 2021).

In the present study, we investigated for the first time the efficacy and safety of TMB-NLC as a preemptive effect for alleviating the severity of acute colitis, given the current difficulties in sufficiently controlling the disorder pharmacologically. Further bioequivalence tests in human subjects will be performed for the development of a novel colon targeted TMB-loaded NLC.

## Materials

Trimebutine maleate was kindly obtained from the National Organization of Drug Control and Research (NODCAR) (Cairo, Egypt). Glyceryl monostearate pellets (m.p. 57–65 °C), Compritol 888 ATO (glyceryl behenate) pellets (m.p. 65–77 °C), Precirol ATO 5 pellets (m.p. 50.0–60.0 °C), Peceol TM (glyceryl monooleate), Miglyol® 812 N, Capryol 90, Labrafac PG (propylene glycol dicaprylocaprate), Gelucire 43/01 were obtained as a gift from Gattfosee Co. Saint-priest, Cedex, France. Poloxamer188 (pluronic F-68) M Wt. 7680–9510 Da was purchased from BASF Corp. (Ludwigshafen, Germany). Tween^®^ 80 polysorbate, sodium chloride (NaCl), glacial acetic acid, castor oil, and stearic acid were kindly provided by Adwic, El Nasr, Pharmaceutical Chemicals Co. (Cairo, Egypt). Absolute ethyl alcohol was obtained from Fisher Scientific Co. (Schwerte, Germany). Oxidative stress markers assay kits were obtained from Biodiagnostic Co. (Giza, Egypt). Tumor necrosis factor-α antibodies were procured from Novus Biologicals (Centennial, CO). Other materials were of analytical grade.

### Lipid screening test

The solubility of TMB was screened in diverse solid lipids which were glyceryl mono stearate (GMS), Compritol 888 ATO, Labrafac^®^ PG, Gelucire 43/01, and Precirol ATO 5. First, an accurate weight (2 g) of each solid lipid was melted at 10 °C above the corresponding melting point in a thermostatically controlled water bath (Vieira et al., 2020). An excess amount (50 mg) of TMB was disseminated in screw-capped tubes containing the liquid lipids (5 mL each) (Miglyol^®^ 812 N, Capryol 90, Castor oil, and Peceol TM) and magnetically swirled for 48 hours at 25 °C.

Then, the samples were centrifuged for 30 min at 10,000 rpm, filtered, and 0.5 mL of the clear supernatant was suitably diluted with ethanol and analyzed spectrophotometrically using Ultraviolet-Visible spectrophotometer: model UV-1601 PC (Shimadzu, Kyoto, Japan). Each determination was performed in triplicate. The most solid and liquid lipid that solubilized the drug was selected for the preparation of TMB-NLC.

### Preparation of TMB-NLC

NLC containing TMB was prepared by the hot homogenization method trailed with an ultra-sonication step. Based on the lipid selection test, primarily the preselected solid lipid was melted at about 60–70 °C, then the liquid lipid was added followed by the addition of TMB. The aqueous phase containing 1 M NaCl and the surfactant blend (Poloxamer 188 and Tween 80) was maintained at the same temperature as the lipid phase and added dropwise to the lipid melt and mixed by magnetic stirring. After that, homogenization of the mixture with an ultrasonic homogenizer (model VC505, Sonics & Materials, Inc., Newtown, CT) was essential for 2 min in ice bath at 90% amplitude (one pulse on and one pulse off) permitting the formation of pre-emulsions. In order to obtain TMB-NLC, the resultant lipid dispersion was cooled down to room temperature. Blank NLC dispersions were organized precisely in the same manner but without the addition of the drug. A summary of the composition of the prepared NLC is specified in Table 1.

**Table 1.** Coded independent variables and properties of TMB-loaded NLC prepared according to 4^2^ full factorial design.

Formula code	Independent variables		Dependent variables			DLE%
	Code of (A)	Code of (B)	MPS (nm)	PDI	EE (%)	
F1	–1	–1	489.13 ± 42.70	0.396 ± 0.025	29.40 ± 2.85	0.86 ± 0.02
F2	–1	–0.3	409.53 ± 19.64	0.486 ± 0.057	27.93 ± 1.89	0.90 ± 0.03
F3	–1	+0.3	545.60 ± 21.77	0.250 ± 0.023	37.01 ± 3.52	1.10 ± 0.05
F4	–1	+1	559.76 ± 56.27	0.413 ± 0.086	29.62 ± 3.12	0.87 ± 0.01
F5	–0.3	–1	261.36 ± 10.43	0.266 ± 0.055	39.64 ± 4.56	1.17 ± 0.04
F6	–0.3	–0.3	339.60 ± 21.70	0.312 ± 0.030	51.70 ± 3.70	1.57 ± 0.03
F7	–0.3	+0.3	431.83 ± 11.38	0.471 ± 0.021	44.28 ± 4.34	1.46 ± 0.06
F8	–0.3	+1	448.53 ± 37.24	0.228 ± 0.025	26.57 ± 2.73	0.81 ± 0.02
F9	+0.3	–1	329.80 ± 2.98	0.403 ± 0.035	62.32 ± 3.33	1.54 ± 0.04
F10	+0.3	–0.3	407.36 ± 4.68	0.162 ± 0.004	55.40 ± 1.77	1.44 ± 0.03
F11	+0.3	+0.3	569.70 ± 7.07	0.253 ± 0.027	43.63 ± 3.57	1.00 ± 0.02
F12	+0.3	+1	440.76 ± 33.40	0.474 ± 0.041	37.52 ± 2.90	0.77 ± 0.01
F13	+1	–1	908.21 ± 68.31	0.595 ± 0.077	50.15 ± 3.24	1.11 ± 0.08
F14	+1	–0.3	609.60 ± 23.56	0.247 ± 0.028	53.12 ± 2.34	1.18 ± 0.10
F15	+1	+0.3	479.10 ± 9.34	0.246 ± 0.015	49.61 ± 6.73	0.98 ± 0.01
F16	+1	+1	357.23 ± 17.64	0.487 ± 0.073	62.77 ± 3.36	1.37 ± 0.04

MPS: mean particle size; PDI: polydispersity index; EE: entrapment efficiency; DLE: drug loading efficiency.

### Lyophilization of TMB-NLC

NLC dispersions were lyophilized using a lyophilizer (Labconco (LYPH.LOCK 4.5), Kansas City, MO) after pre-freezing for 24 h. Pre-optimization for various parameters such as freeze drying periods, temperature, and vacuum conditions is a prerequisite to entirely collect the dried nanoparticles. The vacuum was kept constant at 100 mTorr.

### Design of the experiments and statistical analysis

A complete 4^2^ factorial design (four-levels and two-variables) was utilized to study the outcome of the ratio of liquid to solid lipid and surfactant concentration on the particle size, PDI, and EE% of TMB. Table 2 summarizes the independent variables along with their levels. The model was evaluated in terms of statistical significance by means of analysis of variance (ANOVA) by Design Expert v.12 (Stat-Ease, Inc., Minneapolis, MN).

**Table 2.** The independent formulation variables and their levels applied in the design expert.

Factor	Name	Type	Low actual level	Sub-medium actual level	Medium actual level	High actual level	Low coded level	Sub-medium coded level	Medium coded level	High actual level
*A*	Ratio and total amount of lipid mix	Numeric	1:1 (300 mg)	2:1 (300 mg)	1:1 (450 mg)	2:1 (450 mg)	–1	–0.3	+0.3	+1
*B*	Surfactant concentration	Numeric	0.5%	1%	1.5%	2%	–1	–0.3	+0.3	+1

The surface-response plots and contour plots were investigated by maintaining each factor at its lowest, medium, and highest values while fluctuating the other factor over the study's range. Design Expert v.12 (Stat-Ease, Inc., Minneapolis, MN) was used to solve the experimental designs and polynomial models. The amount of TMB (10 mg) and the ratio of Poloxamer 188 to Tween 80^®^ (2:1) were kept constant and carefully selected based on pre-formulation studies. The coefficient estimate represents the expected change in response per unit change in factor value when all other factors are kept constant (Petkar et al., 2018). Hence, the values of the coefficients, as well as the extra positive (synergistic consequence) or negative (antagonistic consequence) sign, are critical to understand the outcomes.

The following is the complete polynomial regression equation:

(1) $$Y=b0+b1\;A\;+b2\;B+b3\;AB+b4\;A^{2}+b5\;B^{2}+b6A^{2}B+b7{AB}^{2}+b8\;A^{3}+b9B^{3}$$

For this design, the ratio of liquid to solid lipid and total lipid amount *A* with four levels (1:1 at 300 mg, 2:1 at 300 mg, 1:1 at 450 mg, and 2:1 at 450 mg) and emulsifier concentration *B* with four levels of (0.5%, 1%, 1.5%, and 2% w/w) were designated as two critical process parameters (CPPs) (independent variables) (Table 2). Four variables were selected to follow their influence upon particle size, particle distribution, and EE%.

### Characterization of TMB-NLC

#### Particle size analysis

Particle size and polydispersity index (PDI), which determine the degree of the distribution of nanoparticles. They were assessed via a laser scattering particle size distribution analyzer (Zetasizer, Nano-ZS 90, Malvern, Worcestershire, UK). Analysis is performed after the dilution of NLC dispersions with deionized water in ratio of 1:10, bath sonication for 30 s. Three investigations for each sample were made and the mean was recorded.

### Assessment of TMB entrapment efficiency percent

The TMB-NLC dispersion was consistently stirred over a magnetic stirrer for 1 h before being centrifuged for 90 minutes at 13,000 rpm in a high-speed cooling ultracentrifuge (CE16-4X100RD, ACCULAB^®^, Central Islip, NY). The obtained cake was collected, diluted with ethanol, and spectrophotometrically recorded at 268 nm (Shimadzu, model UV-2450, Kyoto, Japan). The percentage of EE was computed by means of the subsequent equation:

(2) $$\mathrm{EE}\%=\frac{\text{amount of entrapped TMB}}{\text{total amount of TMB}}\;\times \;100$$

### Drug loading efficiency percent (DLE%)

It can be calculated by the amount of total entrapped drug divided by the total nanoparticle weight. It is a reflection of the amount of drug delivered per amount encapsulated.

(3) $$\mathrm{DLE}\%=\frac{\text{mass of the drug}}{\text{total mass of the drug loaded nanoparticles}}\;\times \;100$$

### Characterization of the optimized TMB-NLC

#### Measurement of zeta potential of optimized formula

The zeta potential (ZP) of the optimized NLC (F9) dispersion was measured at 25 °C and a 40 V/cm electrical field (Zetasizer Nano ZS; Malvern, Worcestershire, UK). The measurements were repeated three times.

#### Surface morphology

Transmission electron microscopy (TEM, Model JEM-1230, JOEL, Tokyo, Japan) and scanning electron microscopy (SEM, JSM-6510LV, Oxford Instruments, X-Max^N^, Abingdon, UK) were used to visualize the morphology and the external surface characteristics of the optimized NLC formulation (F9) (Garg et al., 2021). For TEM, a few drops of sample dispersion were deposed on formvar/carbon-coated copper grids, followed by the addition of 2% w/v phosphotungstic acid. The grid was air-dried after 3 min incubation at room temperature. The images were processed at 80 kV using iTEM software.

For SEM, the suspended NLC was mounted on a carbon double-adhesive layer on a metal holder and coated with a thin layer of gold. Then, the NLC was scanned at an accelerating voltage of 15 kV (Jeol JSM-5400 LV, Jeol, Tokyo, Japan).

#### Fourier transform infrared spectroscopy

An FTIR spectrophotometer (Thermo Fisher Scientific, Inc., Waltham, MA) was employed to eliminate the likelihood of any interactions among TMB and the designated excipients, viz., liquid and solid lipids used for the preparation of NLC, their physical mixture (PM), plain F9, and F9. The frequency range was 4500–500 cm^–1^ at a spectral resolution of 4 cm^–1^. Each sample was dripped with dry KBr powder and pressed into 1 mm pellets for FTIR measurement (Pereira et al., 2019).

#### Thermal analysis

The thermal characteristics of pure TMB, GMS, PM, plain F9, and F9 were investigated using a differential scanning calorimeter furnished with a thermal analysis data system (DSC-823, Mettler Toledo, Columbus, OH; differential scanning calorimeter, Pyris 6 DSC, Perkin Elmer, Waltham, MA). The PMs were prepared by physically mixing each drug with polymers at the same weight ratio used in the prepared formulation. Briefly, each sample (about 4 mg) was placed into a sealed aluminum pan and double pierced. Thermal scans were recorded over the 25–450 °C temperature range under a continuous nitrogen purge (20 mL/min) at a scanning rate of 10 °C/min. An empty aluminum pan was utilized as a blank.

#### Powder X-ray diffraction

PXRD patterns were attained to assess the crystallinity of the drug and other excipients using an X-ray diffractometer (Rigaku Denki, Rint-2500VL, Tokyo, Japan). Using a Cu-K source (*λ* = 1.542 Å) generated at 30 kV at room temperature, each sample was scanned in the 2*θ* range of 3–45°. NLC formulations with and without TMB were freeze-dried for PXRD analysis. Each sample was loaded on the sample holder and pressed with a glass slide to get a homogenous and flat surface with a scanning rate of 1.5°min^–1^. Of note, DSC and XRD analyses of the liquid lipid (CAP 90) were not performed because of their liquid state (Shi et al., 2013).

#### NLC formulations stability study

A stability study was carried out on freeze-dried optimized TMB-NLC as per ICH guidelines with negligible adjustments (Varela-Fernández et al., 2022). The developed formulation (F9) was lyophilized, sealed in a glass vial, and placed at room temperature (25 °C) for 90 days. The appearance, PS, PDI, and EE% of samples were evaluated regularly at predefined storage time intervals, i.e. 0, 30, 60, and 90 days, following reconstitution. These parameters were assessed at the beginning and end of the stability period performing every measurement in triplicate.

#### *In vitro* drug release study

The *in vitro* drug release study is beneficial for resolving quality issues and predicting TMB-NLC *in vivo* release behavior of TMB from both TMB solution and NLC that was permitted to progress for 12 h using a USP basket tablet dissolution tester (Abbott, Abbott Park, IL) each having a 250 mL capacity. Certain weight of the freeze-dried powder of F9 equivalent to 3 mg TMB was loaded into the donor compartment. The receptor compartment was filled with an HCl solution of pH 1.2 for the first two hours of the release experiment, then gradually pH values were changed and overall transit time was equivalent to the normal distinctions along the GIT. The pH was raised to 6.8 (2–6 h) via adjustment with phosphate buffer and 2 N NaOH (Gao et al., 2021). Afterwards, the pH was increased to 7.4 (6–12 h) to mimic the ileum and colon by the extra addition of 3 mL of NaOH. The content of the cell was constantly agitated at 100 rpm using dissolution apparatus at a constant temperature (37.0 °C ± 0.5 °C). At 0.5, 1, 2, 4, 6, 8, 10, and 12 h, samples of 2 mL were withdrawn from the medium in the receptor compartment by a syringe and replaced with fresh phosphate buffer. The samples were analyzed using UV-Spectrophotometer at *λ*_max_ 268 nm. Each experiment was carried out in triplicate, and the cumulative TMB released (%) at each time interval was calculated.

### Release data analysis

With the aim of describing the release kinetics of the drug from NLC, different kinetic equations, such as zero order, first order, Higuchi’s diffusion, and Korsmeyer-Peppas semi-empirical models, were used to numerically trim *in vitro* release records. The kinetic release profile that assigns the highest coefficient of determination (*R*^2^) was selected to determine the superior mathematical model by means of GraphPad prism software version 6 (La Jolla, CA).

### *In vivo* studies: assessment of ulcerative colitis

#### Experimental animals

Sprague Dawley rats weighing 200 ± 20 g were applied in the UC model. The animal handling and procedures were done according to US National Institute of Health Guide for the Care and Use of Laboratory Animals (NIH publication no. 85-23, revised 1996). The protocol was approved by the Faculty of Pharmacy's Ethical Committee, Mansoura University, Mansoura, Egypt (code no. 2021-383, approval date: November 2021). Animals were kept at a temperature of 25 ± 1 °C, relative humidity of 55 ± 5% and a regular 12 h light/12 h dark cycle. The animals had free access to a standard laboratory diet with water *ad libitum*.

#### Treatment protocol

Twenty-four rats were randomly divided into four groups (six for each group). Rats of group I (normal control) and group II (positive control) were administered normal saline only (10 mL/kg) for five days. Rats of pretreated groups were orally administered; free TMB solution at a dose of 30 mg/kg (TMB group) and TMB loaded NLC (F9) at a dose equivalent to 30 mg/kg TMB (TMB-NLC group). All treatments were administered orally via intragastric tube once daily for five successive days and the last dose was administered 2 h before colitis induction.

#### Induction of acetic acid ulcerative colitis

On the fifth day, all rats were fasted for 16 h, partially anesthetized using chloral hydrate (400 mg/kg, IP) and colitis was induced in all groups except the normal group with 2 mL of acetic acid (4% v/v, in normal saline) injected intra-rectally through a polyethylene catheter in way that the tip was 8 cm inside the anus. To avoid solution leakage, the rats were held vertically in a head-down position for 60 seconds after acetic acid was injected into the rectum (Rashidian et al., 2016).

#### Tissue collection and preparation

After a day of colitis induction, the rats were euthanized by dislocating the cervical vertebrae, and the colon was excised, split longitudinally, washed with normal saline, and the wet weight was measured (Rashidian et al., 2016). A small portion of the final 8 cm of the colon was kept in 10% formalin solution for histopathological evaluation as well as immunohistochemical assessment and the remaining portion was stored at –80 °C for the biochemical assay of oxidative stress markers (glutathione (GSH), superoxide dismutase (SOD), and malondialdehyde (MDA)).

#### Colitis-macroscopic scoring

The colonic parts were blotted dry and photographed to be examined for mucosal damage by an observer who was unaware of the identity of the samples. Visible signs of colonic damage and injury of each ulcer were scored based on a scoring system ranging from 0 to 4. Scores were assigned accordingly; no macroscopic change (0), mucosal erythema only (1), mild mucosal edema, small erosions or slight bleeding (2), moderate edema, slight erosions or ulcers (3) and severe ulceration, edema and necrosis (4) (Adakudugu et al., 2020; Anwer et al., 2020).

Furthermore, the stool of the rats was evaluated for consistency and occult blood one day before and after acetic acid intrarectal administration (Abdel-Daim et al., 2015). Weight-to-length ratios (g/cm) were calculated by dividing wet weight by colon length to get a measure of wall thickening and edema (Duijvestein et al., 2011).

#### Histopathological examination

Samples of the colon tissues were fixed immediately in 10% formalin, dehydrated, paraffin embedded, cut into sections of 5 μm, deparaffinized with xylene, hydrated, then mounted and stained with hematoxylin and eosin (H&E) as well as Alcian blue special stain. The stained sections were examined for the histopathological changes of colonic tissues under a light microscope (Leica Microsystems, Wetzlar, Germany) by a skilled pathologist who is not aware of the identification of the specimens to avoid any bias.

A grading scoring system ranging from 0 to 11 was used to grade microscopic alterations (Appleyard & Wallace, 1995; Adakudugu et al., 2020). Scores were assigned accordingly: loss of mucosal architecture (0–3), cellular infiltration (0–3) muscle thickening (0–3), crypt abscess formation (0–1), and goblet cell depletion (0–1); for scales (0–3), none (0), mild (1), moderate (2), and severe (3), as well as, for scores (0–1), negative (0), and positive (1).

#### Immunohistochemical localization of TNF-α

Another set of sections was subjected to immunohistochemical staining using monoclonal antibodies targeting TNF-α employing the procedure as previously reported (Kotakadi et al., 2008; Schreiber et al., 2013).

A digital camera (Olympus Corporation, Tokyo, Japan) mounted on a light microscope (Leica Microsystems, Wetzlar, Germany) was used to measure the intensity of positively labeled cells. The following scale was used to rate the intensity of immunohistochemical staining: 0 indicates no staining, 1 indicates mild staining, 2 indicates moderate staining, and 3 indicates significant staining (Fisher et al., 2005). All readings were blindly done by a qualified pathologist.

#### Assessment of oxidative stress markers

Colonic tissues (0.5 g each) were grinded in a mortar with liquid nitrogen, added 4.5 mL of phosphate buffer (pH 7.4), homogenized on ice and centrifuged at 4 °C (Heraeus, GmbH, Osterode, Germany) (Abd El Hady et al., 2021). The supernatants were analyzed to estimate the levels of colonic GSH, SOD, and MDA via their appropriate commercially available kits, in accordance with the manufacturer's instructions.

### Statistical analysis

Statistical analysis using one-way ANOVA, accompanied with Tukey–Kramer multiple comparisons test was achieved by GraphPad Prism version 6.00 (GraphPad Software, Inc., La Jolla, CA). The statistical differences at *p*<.05 were deliberated as significant.

## Results and discussion

### Lipid screening test

**Figure 1.**
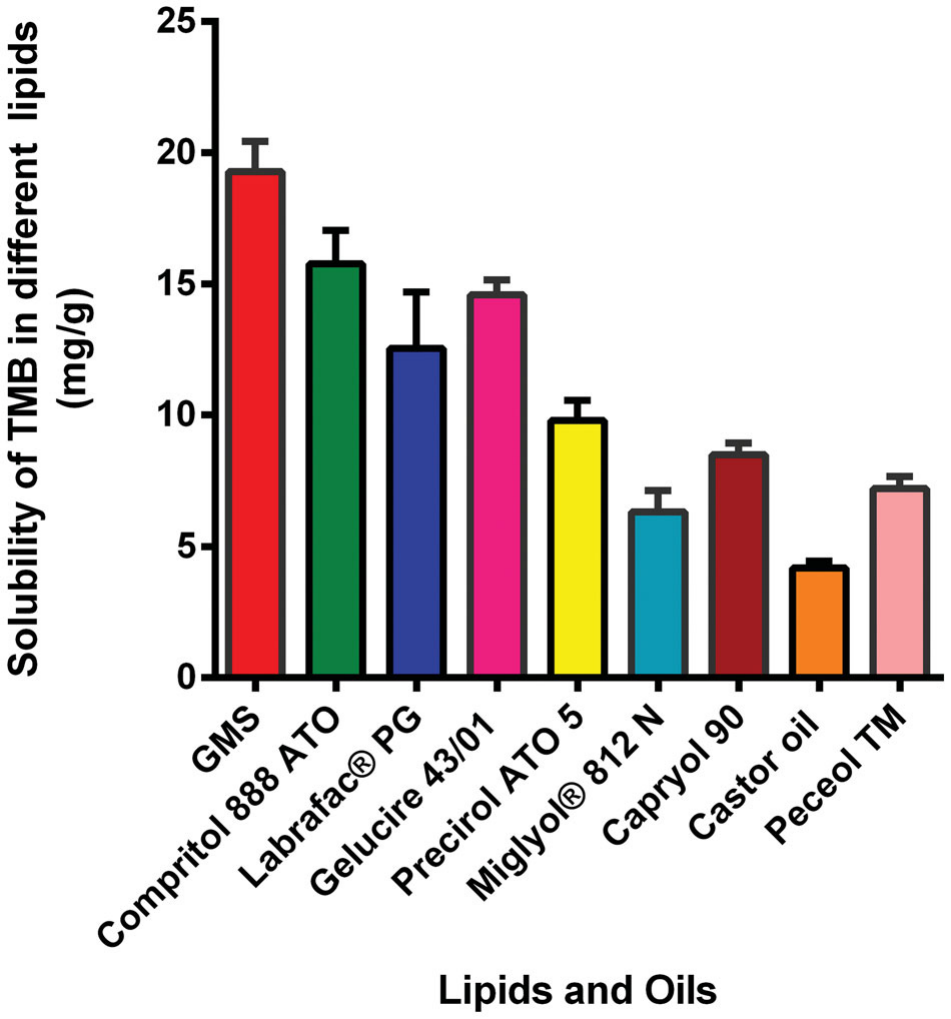
Solubility profile of TMB in solid and liquid lipids.

Solubility of the drug in the melted lipid is recognized to be an essential precondition to achieve sufficient EE% (Vitorino et al., 2013). Glyceryl monostearate exhibited the highest solubilizing capacity compared to the other solid lipids tested. In the case of liquid lipids tested, TMB was most soluble in CAP 90 as shown in Figure 1. Consequently, they were selected for the preparation of TMB-NLC.

### Preparation of TMB-loaded NLC

Factorial design is a multivariable data analysis procedure that can be used to adjust the manufacturing processes of lipid nanocarriers via hot homogenization, affording valuable data and reducing the progress time. In our study, we described how to use factorial design to optimize a process for the preparation of TMB-NLC based on 300 mg or 450 mg of lipid content and Tw 80 with P 188 as the colloidal stabilizers to form the NLC dispersion in 16 different formulations (Table 1). As aqueous NLC dispersion is prone to physical and chemical stability complications, freeze-drying is regarded as the primary solution for improving NLC stability for long-term storage through their incorporation into solid dosage forms.

### Experimental design

#### Effect of formulation variables on particle size of NLC

Regression coefficients for the responses *Y*1 (mean particle size) was revealed in the following equation:

(4) $$\mathrm{MPS}\;(Y1)\;=+\;385.17+\;102.33\;A\;+233.63\;B\;–148.03\;AB\;+159.14\;A^{2}+0.79\;B^{2}\;–\;217.55A^{2}B+7.79{AB}^{2}\;–63.33\;A^{3}\;–135.14\;B^{3}$$

It is possible to use the equation in terms of coded factors to create estimates regarding the response at various degrees of each element. Moreover, it is also useful for detecting the comparative impact of the factors by matching the factor coefficients. From the model summary statistics, we found that the model was best fitted with the ‘Cubic model’ which maximizes the adjusted *R*^2^ and the predicted *R*^2^.

It was perceived that particle size was proportional to total lipid ratio and concentration, as well as surfactant concentration. This increase in the mean particle size of lipid nanoparticles could be attributed to the increasing viscosity of the oil phase (Teeranachaideekul et al., 2008). Also, because of the larger liquid lipid content, the viscosity inside NLC was lowered, enhancing their fluidity and, as a result, the surface tension was reduced, resulting in smaller and smoother surface particles (Sanad et al., 2010; Andalib et al., 2012).

**Figure 2.**
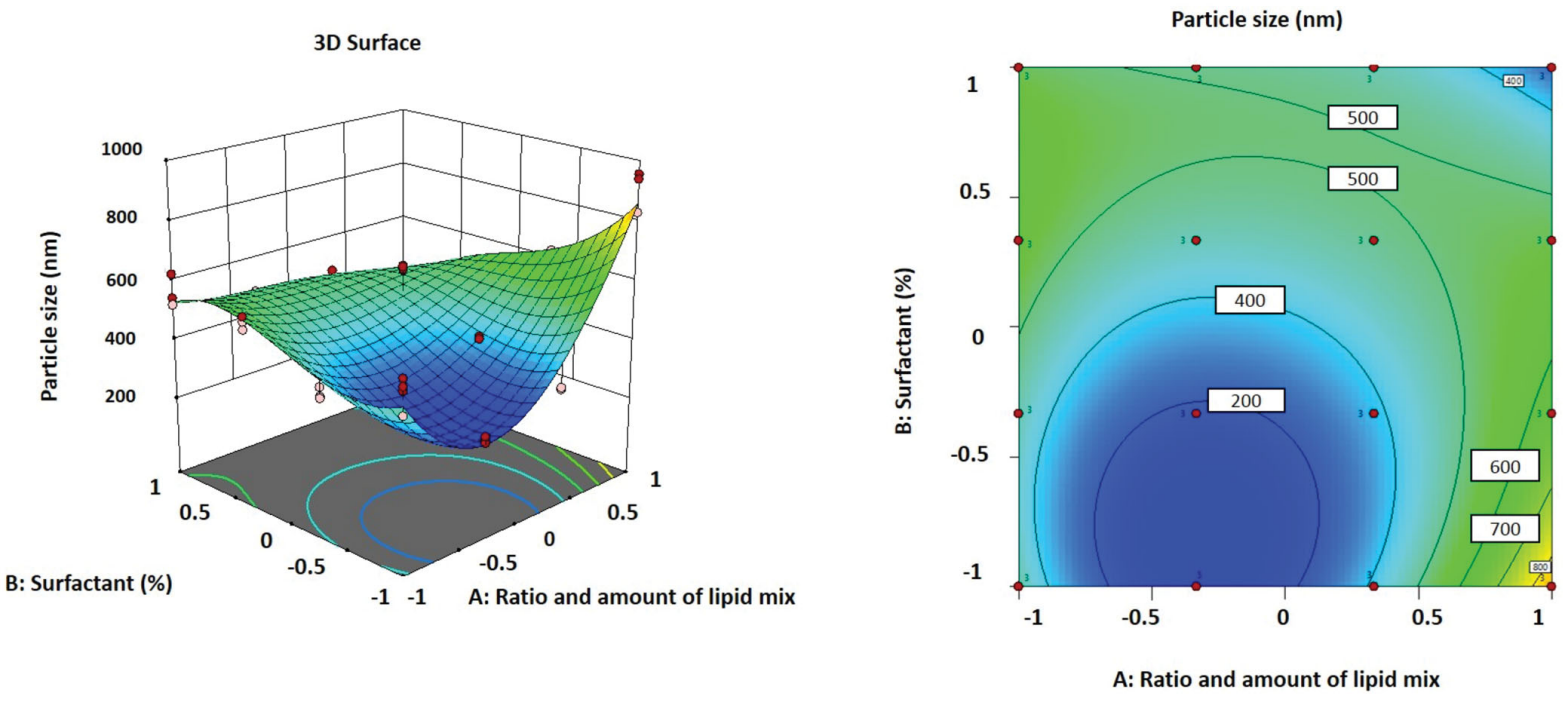
Three-dimensional surface plot (A); contour plot (B) illustrating the influences of independent variables *A* and *B* on particle size.

The relationship between variables was additionally interpreted using contour and three-dimensional surface plots (Figure 2).

The lowest particle size was found in formulation F5, which had a low value of the independent variables, which could be attributable to the minimum concentration of the lipid blend.

In Table 1, the particle size values for all batches of TMB loaded with NLC are shown for all batches. The different quantities of independent variables in the formulation resulted in particle sizes ranging from 261.36 ± 10.43 nm to 908.21 ± 68.31 nm for all formulations. The particle size data revealed that when the amount of liquid lipid in the formulation augmented, the particle size increased.

#### Effect of formulation variables on PDI

Regression coefficients for the responses *Y*2 (PDI) are shown in the following equation:

(5) $$\mathrm{PDI}\;(Y2)\;=\;+\;0.246\;–\;0.11\;A\;+\;0.04\;B\;–\;0.001\text{ AB }+\;0.085\;A^{2}\;+\;0.11\;B^{2}\;–\;0.08\;A^{2}B+0.19\;{AB}^{2}+0.02\;A^{3}\;–0.001\;B^{3}$$

It was observed that particle distribution was inversely proportional to the ratio and concentration of total lipid, but it was directly proportional to the concentration of surfactant. As the surfactant concentration increased from 0.5 to 2% w/v, the interfacial tension among the lipid phase and aqueous phase decreased, allowing for better stabilization of the smaller lipid droplets. That was performed through the creation of a steric barrier on the particle surface, which thus guards smaller particles and prevents them from coalescing into larger ones. Consequently, a stable emulsion with smaller and uniform droplet size nanoparticles and a low PDI may be exhibited (Kovacevic et al., 2011; Moghddam et al., 2017; Emami et al., 2018).

**Figure 3.**
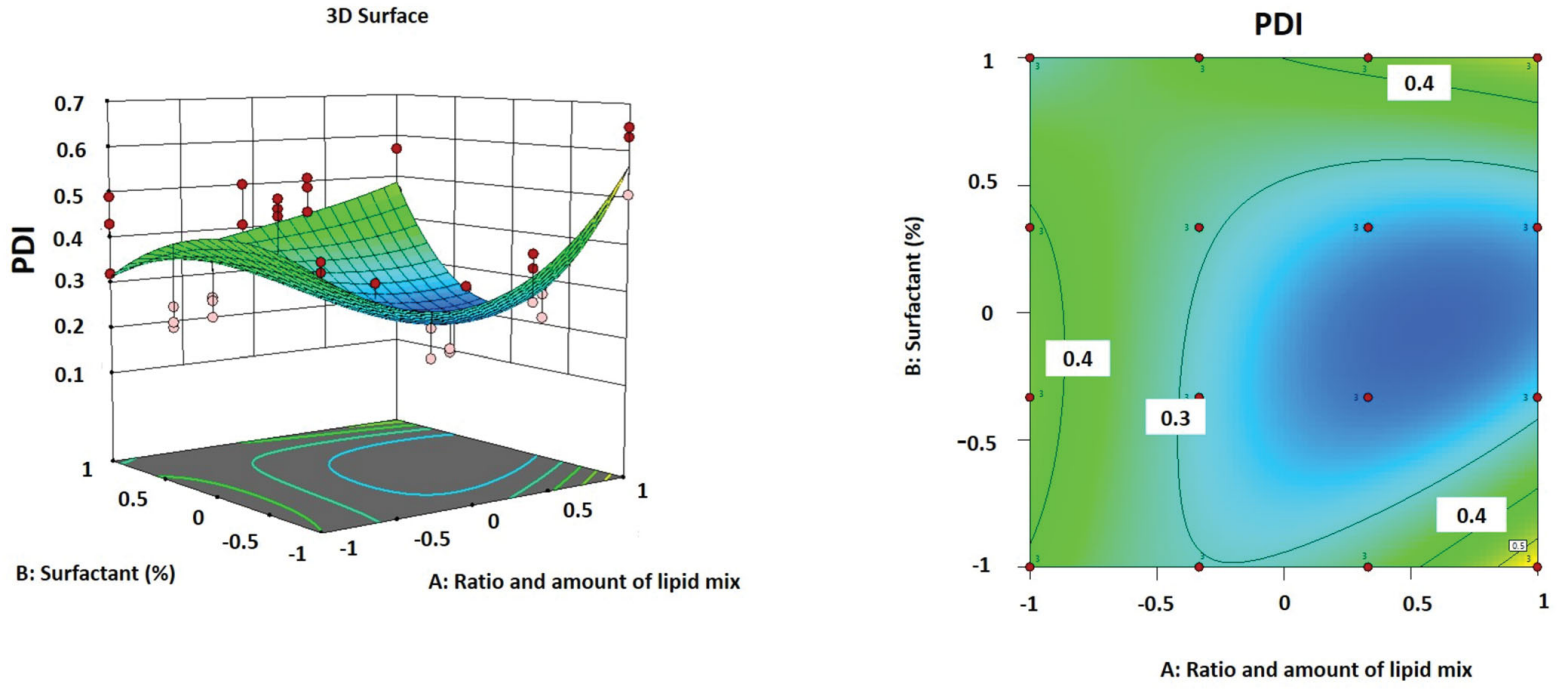
Three-dimensional surface plot (A); contour plot (B) illustrating the influences of independent variables *A* and *B* on PDI.

The correlation between variables was supplementary clarified using contour and three-dimensional surface plots (Figure 3).

#### Effect of formulation variables on EE% and drug loading efficiency percent

**Figure 4.**
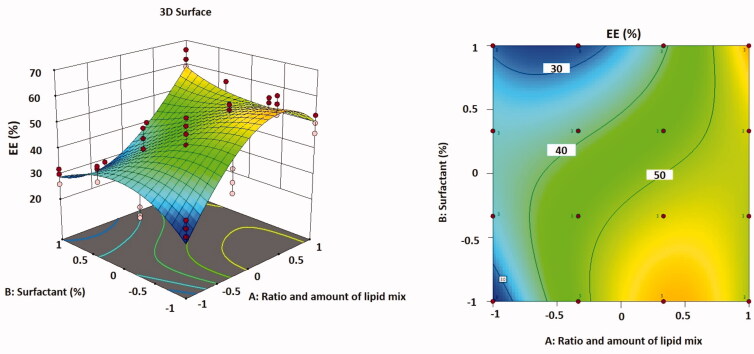
Three-dimensional surface plot (A); contour plot (B) illustrating the influences of independent variables *A* and *B* on EE%.

In Equation (6), regression coefficients for the responses *Y*3 (EE%) are revealed. The EE% improved reasonably as the total lipid concentration declined and the proportion of liquid lipid increased. A maximum value of EE% was detected in optimized formulation F9. The association between variables was further simplified using contour and three-dimensional surface plots (Figure 4).

(6) $$\text{EE\% }(Y3)\;=\;+\;46.90\;+\;10.54\;A\;–13.25\;B\;+\;0.78\text{ AB }–\;2.86\;A^{2}\;–\;3.52\;B^{2}+14.93\;A^{2}B+6.55\;{AB}^{2}–2.77\;A^{3}+1.83\;B^{3}$$

The EE% and DLE% are chiefly reliant on the nature of the drug and the lipid phase in which it is encapsulated. As TMB is a hydrophilic drug, the EE% was found to be relatively high, in the range of 26.57–62.32%, and the DLE% was found to be in the range of 0.77–1.57%, as shown in Table 1. It was experimentally detected that the particle size growth during centrifugation has risen as a result of the addition of NaCl, and accordingly, the efficacy of centrifugation as well as the entrapment of the medication inside NLC. This was deduced from the fact that if the concentration of NaCl was high enough (>50 mM), it had a considerable effect on the production of vesicles, which was predicted to be due to its osmotic pressure (Sut & Jackman, 2019).

It was also thought that increasing the oil content in NLC increased the percentage of encapsulated medicine because drug solubility in the liquid lipid was higher than in the solid lipid. This could explain why NLC has a greater EE% of TMB (Patil et al., 2016). Another explanation of such increased EE% that, higher liquid lipid content into the solid lipid matrix (GMS) could increase the solubility of the drug in the lipid matrix, which increases its EE% (Müller et al., 2002). The high EE% values observed in this study specified that the used lipids and surfactants were suitable for TMB entrapment in NLC.

Generally, it is indicated that NLC may carry more drugs due to the presence of amorphous liquid lipid, which can distort the perfect crystallization of solid lipids, thus increasing the drug loading capacity (Wu et al., 2021).

ANOVA was performed and selected for cubic model that maximizing the adjusted *R*^2^ and the predicted *R*^2^ wherever the additional terms were significant and the model was not aliased. The regression analysis outcomes for all the three responses (*Y*1, *Y*2, and *Y*3) are précised in Table 3. The ‘Predicted *R*^2^’ values for all the dependent variables were established to be in reasonable agreement with the ‘Adjusted *R*^2^’ values. All *p* values were less than .05 indicating that the model terms were significant.

**Table 3.** ANOVA results for responses; MPS (*Y*1), PDI (*Y*2), and EE% (*Y*3) obtained from experimental design response.

Response	Source	*F* value	*p* Value	Inference	Adjusted *R*^2^	Predicted *R*^2^	Adequate precision
MPS (*Y*1)	Cubic	21.49	<.0001	Significant	0.8539	0.7879	21.46
PDI (*Y*2)	Cubic	4.69	0.0010	Significant	0.4243	0.1843	7.6368
EE% (*Y*3)	Cubic	12.06	<.0001	Significant	0.7990	0.7151	14.34

MPS: mean particle size; PDI: polydispersity index; EE: entrapment efficiency.

In sum, the regression model equations revealed that the positive cubic contribution of (*A*, *B*, *AB*, *A*^2^, *A*^2^*B*, *B*^3^), which was the main model term, had a considerable influence on the response *Y*1 (MPS) while, *Y*2 (PDI) was affected by *A*^2^, *B*^2^, *AB*^2^ terms. The response *Y*3 (EE%) was significantly dependent on the positive effects of *A*, *B*, *A*^2^*B*, *AB*^2^ which were the significant model terms.

ANOVA was used to assess the response surface models. The model value was 0.05, indicating that the predicted model could accurately predict the relationship between the independent and dependent variables.

The signal-to-noise ratio is measured with adequate precision. It is preferable to have a ratio of more than four. The design space can be directed using this concept.

The response surface is based on Design Expert Version 12.0.9.0 (study type). For optimization with the prediction objective fitted at the most lowered (particle size and PDI) and maximized (EE%), use a user defined design type with a desirability factor of 99%. The optimized formula F9 with (*A* [+0.3] and *B* [–1]) represented the highest EE%, moreover low particle size and PDI were achieved. These actual results were in close accordance with the predicted values by the software. The optimized TMB-NLC was organized with the adjusted level of components and the procedure variables are summarized in Table 4.

**Table 4.** Adjusted levels of independent variables and their analogous predicted and observed responses for optimized formula.

Optimized formula coded level	Optimized actual value	Responses	Predicted mean value^a^	Observed experimental value
Ratio and total amount of lipid mix [+0.3]	1:1 (450 mg)	*Y*1 particle size	324.81 nm	329.80 ± 2.98 nm
		*Y*2 PDI	0.487	0.403 ± 0.035
Surfactant concentration [–1]	0.5 (%)	*Y*3 EE (%)	59.15%	62.32 ± 3.33%

PDI: polydispersity index; EE: entrapment efficiency.

^a^ Point prediction at: confidence = 95% and population = 99%.

### Characterization of optimized TMB-NLC

#### Optimized formula zeta potential

The optimized formula (F9) was found to be –24.6 ± 0.8 mV. This may be interpreted as the presence of a carboxylic acid group in oleic acid, which results in a higher negative ZP. Such anionic nano-delivery systems were selectively attached via electrostatic interaction to the positively charged cationic proteins, which increased in the inflamed tissues and consequently sustained the release of the drug from NLC (Hua et al., 2015).

Encouraged by the aforementioned results, it was concluded that TMB-NLC (F9) was a promising delivery system and, hence, it would be worthwhile to undergo further studies.

#### Surface morphology

**Figure 5.**
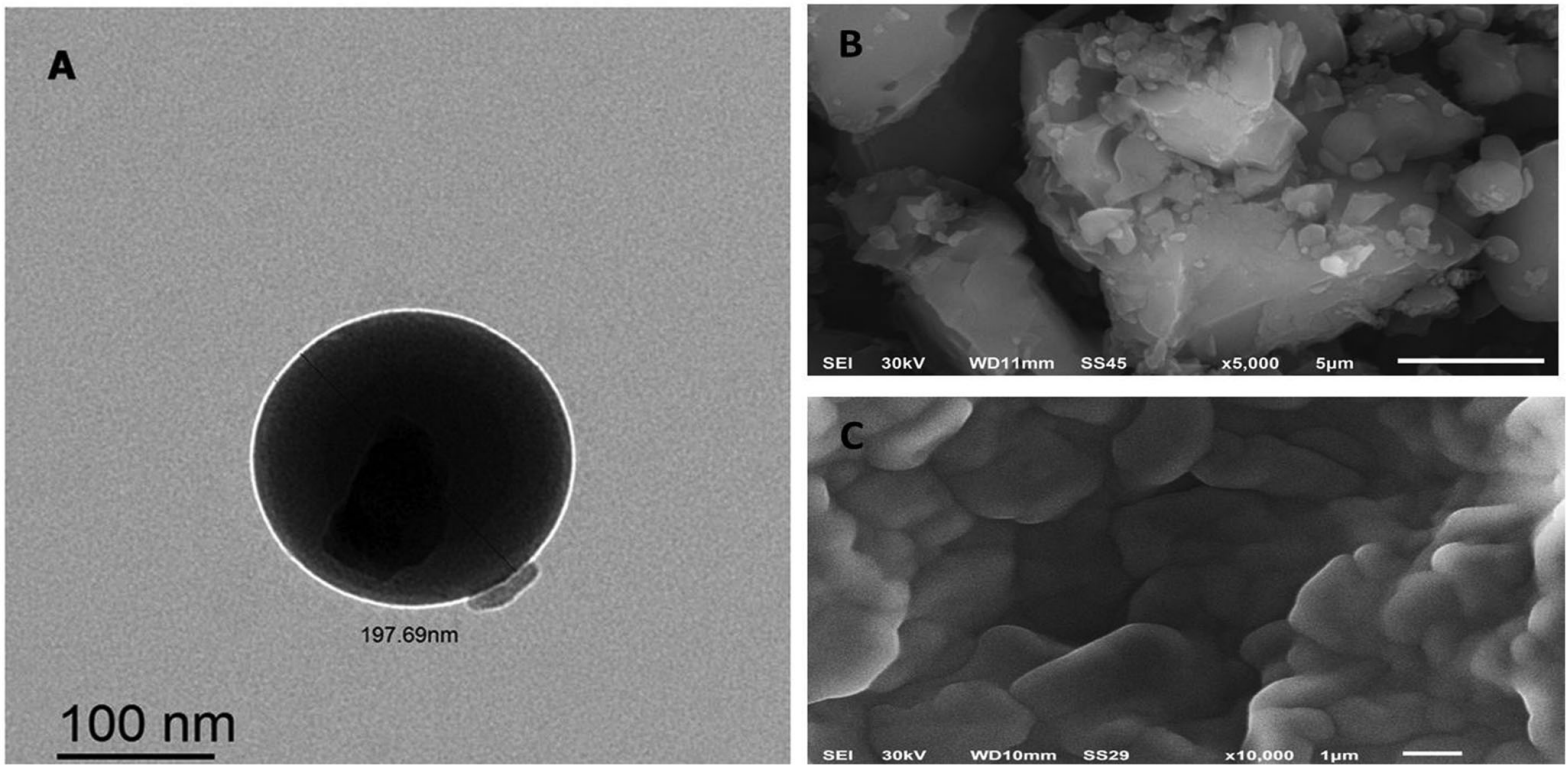
Representative micrographs for morphological examination of F9-TEM (A), TMB-SEM (B), and F9-SEM (C).

The results obtained in particle size analysis of the optimized formulation (F9) were further verified by TEM and SEM (Figure 5). TEM has been broadly employed as a powerful approach for identifying the size and surface morphology of NPs. The TEM images of F9, indicating a small, globular shape with a spherical homogenous lipid core surrounded by a surfactant corona, as depicted in Figure 5(A). Particle agglomeration was not observed for the developed TMB-NLC (Pereira et al., 2019). It can be revealed from the images that PSD appears quite uniform, as evident by the low PDI attained with DLS data. The formation of the NPs stable spherical bilayer may be owing to the reduced surface and minimal interfacial tension provoked by the presence of emulsifiers (Ortiz et al., 2021). Notably, the diameter of the NLC in the TEM images (about 150 nm) was smaller than that measured by DLS (Figure 5(A)). On one hand, this difference may be principally contributed to measuring the hydrodynamic radius by DLS, which encloses the double solvation layer. On the other hand, TEM showed the size of dried samples without surface ions and solvent molecules in contrast to DLS measurements. Comparable elucidations and judgments have been established in the literature (Courant et al., 2009; Ortiz et al., 2021).

SEM is a qualitative process used to study the surface morphology of raw materials (Chao et al., 2016). As displayed in Figure 5(B,C), the drug itself seemed like an asymmetrical-shaped crystal particle. However, the NLC crystallized into a lamellate form, which was quite different from the shape and size of the pure drug. Scanning electron microscopy of the prepared batch showed spherical particles in the nanoscale range, a smooth surface, uniform size, and free from drug crystals, as shown in Figure 5(C). Accordingly, the investigation of particle size by employing SEM was larger than that obtained by DLS analysis. Generally, the nanodispersion is transformed into a powder, mainly by the lyophilization process. Before measurement under high vacuum, these dried NPs are then sputtered with gold as a conductor, providing a coating on the surface, tending to exhibit a larger size than DLS measurements (Khosa et al., 2018).

#### FTIR

**Figure 6.**
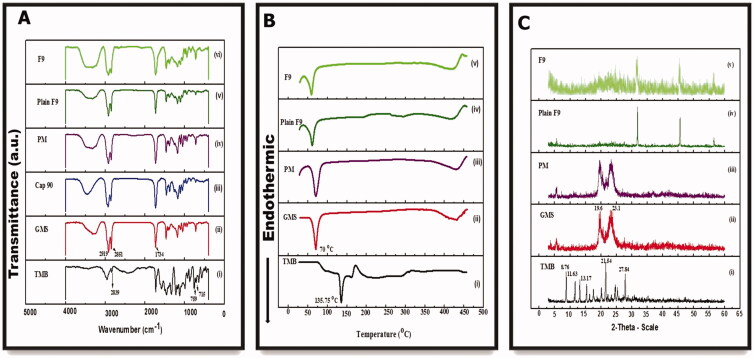
Fourier-transform infrared (A), differential scanning calorimeter (B), and powder X-ray diffraction (C) spectra of (i) TMB, (ii) GMS, (iii) PM, (iv) plain F9, and (v) TMB-NLC (F9).

Infrared spectroscopy is considered a beneficial qualitative means to characterize the interaction between the pharmaceutical system's components (Pereira et al., 2019). This analysis was done on the major ingredients of the prepared NLC, i.e. TMB, GMS, CAP 90, the PM of them, and the two NLC formulations (plain and medicated F9). The IR spectrum of pure TMB (Figure 6(A(i))) exhibited at least three characteristic absorption bands at 711, as well as 759 cm^–1^ (relating to C–H vibration in the mono-substituted benzene ring), and 2839 cm^–1^ (attributed to stretching vibration in N, N-dimethyl C–H) (Chao et al., 2016). As clearly demonstrated in Figure 6(A), the FT-IR spectra of all samples displayed prominent resemblances and overlapped. The high-intensity band observed at 1735 cm^–1^ was assigned to carbonyl (C=O) group vibration. As demonstrated in Figure 6(A(ii)), the high-intensity peaks noticed at 2919 and 2851 cm^–1^ correspond to the C–H stretching vibrations of lipids, characteristic of olefinic double bonds (C=C), and methyl (C–H) groups of fatty acid chains, respectively. The C–O vibration bond of ester bonds present in triacylglycerols is attached to the order of peaks identified at 1259, 1179, 1106, and 1051 cm^–1^ (Pereira et al., 2019). Besides, these characteristic peaks appeared in the FTIR spectra of the NLC formulations because the lipid matrix is the crucial excipient. Additionally, the spectrum of TMB in the NLC spectra has almost completely vanished, implying an effective drug entrapment into the NLC matrix (Pereira et al., 2019).

#### Thermal analysis

The change in thermal behaviors between the drug and the used excipients during the formulation of TMB-NLC can be represented by DSC analysis (Sinhmar et al., 2018). Figure 6(B) shows the thermal behaviors of the drug, polymers, PM, plain F9, and F9. The DSC curve displayed a sharp endothermic melting peak at about 135 °C (Figure 6(B(i))) inconsistent with the data in the literature (Abourehab et al., 2021), which indicates the purity of the drug. The endothermic peaks of GMS were observed at about 70 °C (Figure 6(B(ii))). Furthermore, PM demonstrated no intrinsic peak of the drug (Figure 6(B(iii))). A slight change was observed in the peak of the plain NLC (60 °C) compared to the PM of lipids, attributed to the presence of another component such as an emulsifier and surfactant (Figure 6(B(iv))). Besides, the drug peak was not noticed in TMB-NLC (Figure 6(B(v))); this could be inferred that the incorporated drug may get embedded in a molecularly dispersed form inside the lipid matrix, and no detectable drug crystals are formed during the production of NLC (Sinhmar et al., 2018; Pereira et al., 2019).

#### PXRD

Figure 6(C) shows the PXRD data of TMB, GMS, and a PM of them, the NLC formulation with and without TMB. The diffractograms of the drug showed numerous peaks at 2*θ* 8.76, 11.63, 13.17, 21.55, and 27.84°. The diffraction pattern of TMB crystalline powder is depicted in Figure 6(C(i)). For GMS, the sharp peaks were at 19.6°and 23.1°, which correspond to short spacings at 0.45 and 0.38 nm, indicative of the polymorphic forms *β* and *β*′, respectively, that are strongly related to drug release and incorporation (Pereira et al., 2019) (Figure 6(C(ii))). These forms have highly ordered fatty acid chains (Gu et al., 2022). Although the crystalline peaks of TMB were clearly observed for the PM (Figure 6(C(iii))), they were not observed for the NLC formulations, possibly indicating that the TMB encapsulated in NLC is completely dispersed in an amorphous form (Figure 6(C(v))). Nevertheless, the principal peaks of the involved lipid in the formulation did not shift, but it was less prominent (Hasan et al., 2021). This characteristic is desirable to produce NLC; since a solid fat with a less ordered matrix; diminishes the possibility of stability concerns accompanied by the entrapped drug expulsion during storage (Pereira et al., 2019). This technique is noteworthy because it emphasizes on modifications of crystalline components of the formulations, harmonizing the DSC data.

#### *In vitro* drug release study

**Figure 7.**
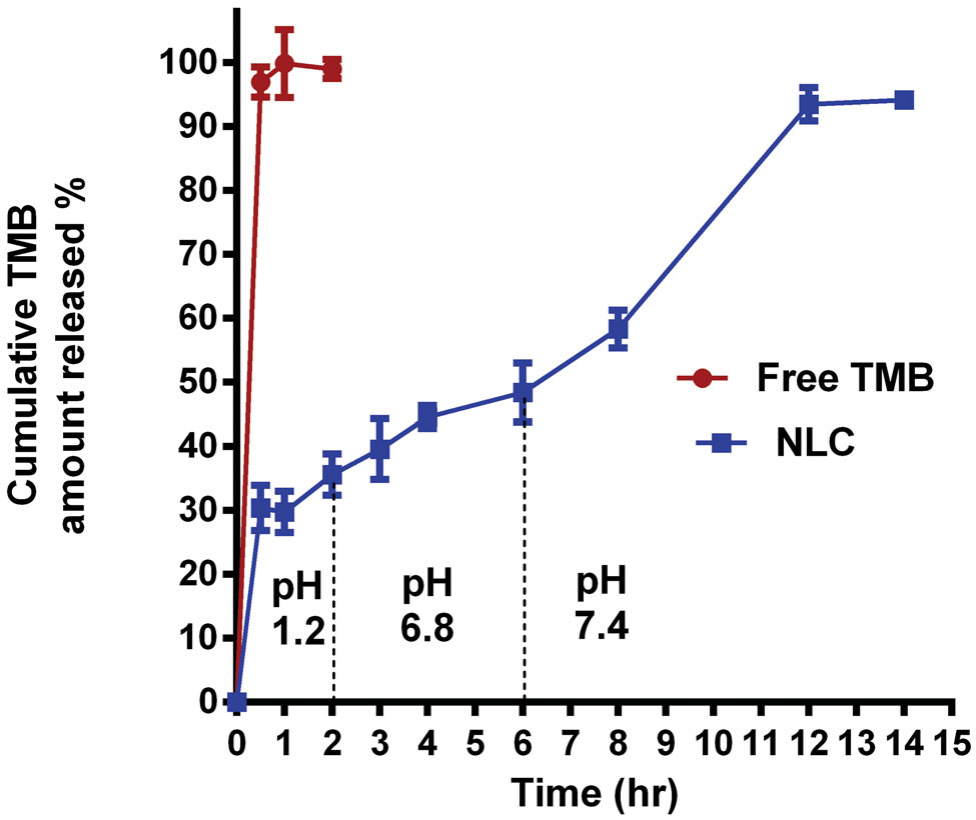
Cumulative released percent of TMB from solution and NLC at different pHs.

To demonstrate the mechanism of TMB release from NLC, *in vitro* release research was done for up to 12 h using dissolution equipment. The *in vitro* release of TMB from the drug solution alone and the chosen TMB-NLC F9 formula is shown in Figure 7.

At pH 1.2, free TMB diffusion was completed at nearly 1 h. In contrast, the release profile of TMB from NLC was more sustained and possessed a biphasic pattern, with a burst and a prolonged drug release, which was necessary to extend the drug's retention time and confirm efficacy. It was fast, nearly 32.08 ± 4.3% of TMB released within two hours, followed by a slower, continuous release of 94.3 ± 3.3% over 12 h. The initial burst effect could be attributed to the short diffusion path of the drug portion on the outer surface of NLC. Whereas, TMB was uniformly distributed and entrapped into the structured lipid matrix during the second phase of delayed release, and it can only be freed from it through gradual dissolution and diffusion.

The results of this research revealed that the *in vitro* drug release kinetics of TMB from NLC closely followed the Higuchi kinetic model (*R*^2^=0.93). The values of the release exponent, n, measured by the Peppas model were found to be 0.24, which elucidated that, the release profile obeyed the Fickian diffusion mechanism (Table 5).

**Table 5.** Kinetic release models of free trimebutine maleate (CL) and NLC-F9 formula.

Formula code	Coefficient of determination			Korsmeyer-Peppas			Main transport mechanism
	Zero-order	First-order	Higuchi model	*R* ^2^	*N*	*K*	
CL	0.48	0.49	0.77	NA	0.01	1.9	NA
NLC-F9	0.92	0.89	0.93	0.83	0.24	1.5	Fickian

CL: trimebutine maleate solution (control); *N*: diffusional exponent indicative to the mechanism of drug release (slope); *K*: intercept; *R*^2^: correlation coefficient; NA: not applicable.

#### Stability studies of TMB-NLC formulations

The effect of storage time on PS, PDI, ZP, and EE% of TMB NLC is a key factor for evaluating the stability of these lipid nanoparticles. After three months of storage at 25 °C, there was a statistically significant difference (*p*<.05) in particle size for TMB-NLC when compared to a freshly prepared formulation. Upon analysis of TMB-NLC formulations after one, two, and three months; it was identified that at 25 °C, PS was significantly increased (*p*<.05) for F9 (1.06, 1.25, and 1.27 times) when compared to the freshly prepared formula (Table 6). Despite these discrepancies, the particle size of NLC remained within an acceptable submicron range (<500 nm). All values of PDI and ZP were within the desired range ≤0.5 and –24 to –34 mV, respectively, and hence not considered significant in the current study (Muhindo et al., 2021).

**Table 6.** Average particle size, polydispersity index, zeta potential, and % entrapment efficiency of the freeze-dried optimized TMB-NLC (F9) formula after storage for three months at 25 °C in comparison to fresh formula (mean ± SD, *n* = 3).

Storage time (months)	PS ± SD	PDI ± SD	ZP ± SD	%EE ± SD
Zero	378.33 ± 6.21	0.22 ± 0.03	‒24.42 ± 0.65	70.17 ± 2.32
First	402.10 ± 5.63*	0.23 ± 0.01	–34.13 ± 0.85*	68.74 ± 3.21
Second	472.30 ± 6.30*	0.28 ± 0.02	‒30.03 ± 0.49*	66.71 ± 1.98
Third	479.5 ± 7.12*	0.27 ± 0.02	‒24.43 ± 0.85	58.77 ± 3.45*

PS: particle size; PDI: polydispersity index; ZP: zeta potential; EE%: entrapment efficiency.

* *p*<.05 significant vs. corresponding values of fresh sample.

Test used: one-way ANOVA followed by post hoc Tukey’s test for data expressed in mean ± SD.

Additionally, there were no instability phenomena during storage, like and color change, aggregation, flocculation, and gelation, attributed to the presence of surfactants. The space repulsion of nonionic surfactants (e.g. Tw 80 and F-68) is the principal interaction among NLC semi-solid particles. From this point of view, the key parameters affecting the emulsifying influence in colloidal systems are their concentration plus their hydrophilic–lipophilic balance (HLB) values. In this context, nanocolloidal particles have a great specific surface area. When the emulsifier amount is inadequate, there will be lipid particles that are not encapsulated inside the NLC, causing gelation, flocculation, and aggregation. Also, these instabilities will be noted if the emulsifier cannot provide the desired HLB required for stabilizing these lipid nanoparticles. After 90 days of storage, greater than 58% of TMB was retained in NLC (Table 6), suggesting that the ratio and concentration of Tw 80 and P 188 had an excellent protecting impact on the drug (Gu et al., 2022). These results signify that TMB NLC has high stability upon storage.

### *In vivo* evaluation

#### Colitis-macroscopic scoring

Acetic acid caused colonic lesion and loose stool with occult blood as well as severe macroscopic edematous inflammation, as assessed by the high macroscopic damage scores and the increased colon weight-to-length ratio. The wet weight of the inflamed colon tissue can be directly correlated with the severity and extent of inflammation (Rashidian et al., 2016). An increase in inflammatory cell infiltration, vascular permeability, goblet cell hyperplasia, and edema of the colon leads to the increase in colon weight-to-length ratio (Owusu et al., 2020).

**Figure 8.**
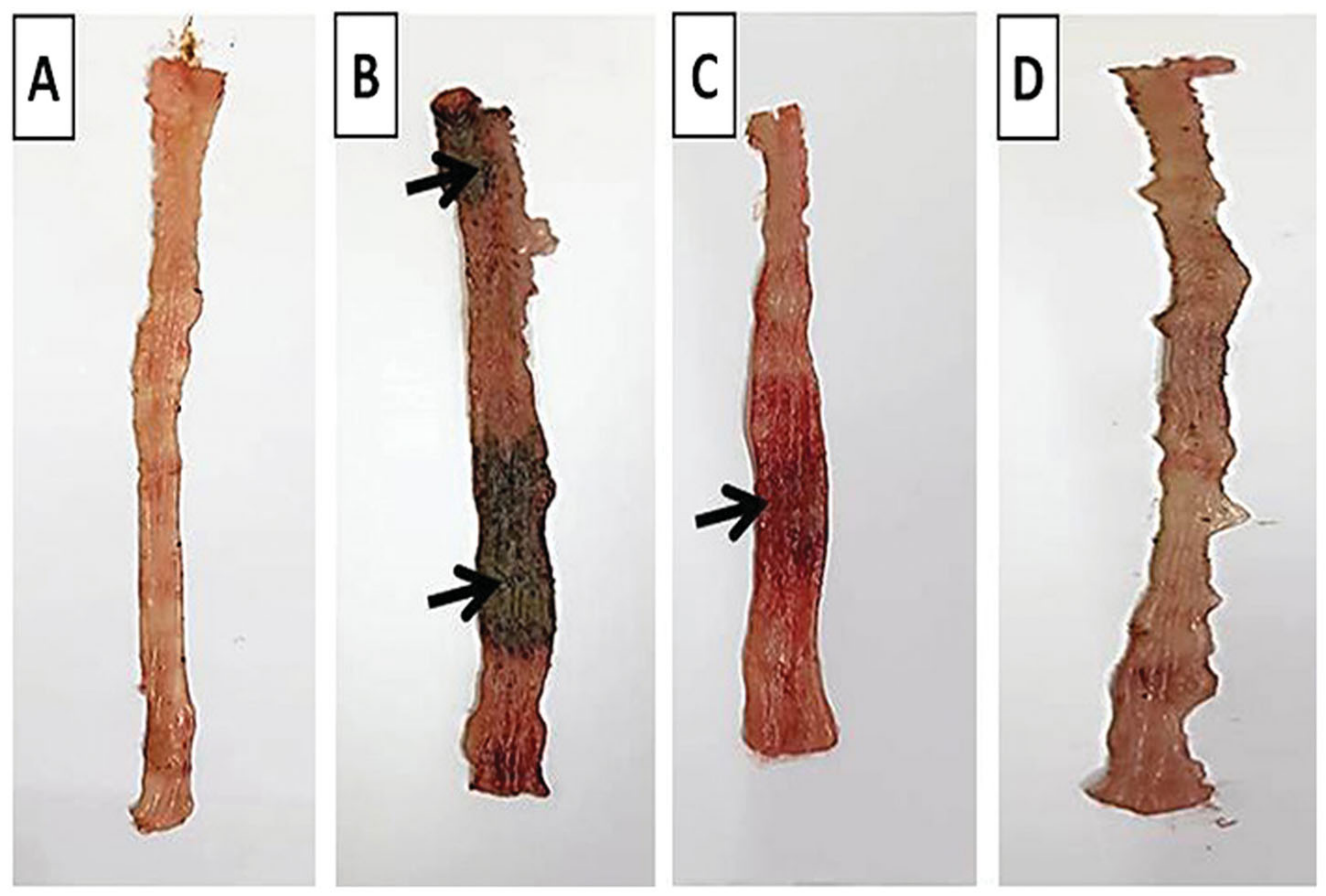
Macroscopic photographs of colon tissue after acetic acid-induced colitis in rats. (A) Normal group; (B) positive control group; (C) TMB group; (D) TMB-NLC group. Macroscopic damage score (black arrow).

The normal group showed intact normal colonic mucosa with no signs of tissue damage (Figure 8(A)). On the other hand, severe ulceration and hemorrhage (black arrow) over a wide surface area of colonic tissue were observed in positive control group (Figure 8(B)). The pretreated group that received free TMB showed moderate damage of rat colonic tissues (Figure 8(C)). Apparently normal colonic mucosa was seen in rats pretreated with TMB-NLC (Figure 8(D)).

**Figure 9.**
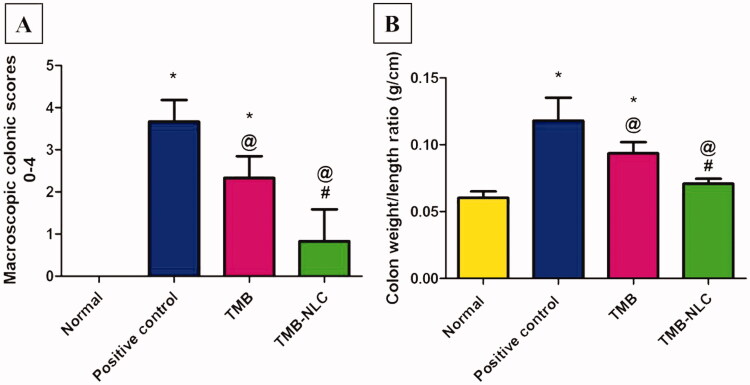
(A) Statistical analysis of macroscopic scores in colonic tissues. (B) Colon weight/length ratio (g/cm) after acetic acid-induced colitis in rats. Data are mean ± SD, *n* = 6; statistical differences at *p*< .05 considered significant; *vs. normal group; ^@^vs. positive control group; ^#^vs. TMB group.

Results of statistical analysis of macroscopic scores of colonic tissues and colon weight/length ratio are depicted in Figure 9(A,B). The highest score was recorded in the positive control group with a high colon weight-to-length ratio. TMB significantly reduced macroscopic damage score and colon weight-to-length ratio in both pretreated rat groups compared to the positive control group. On the contrary, TMB-NLC showed a significant decrease in macroscopic damage scores and colon weight-to-length ratio compared to the free TMB group. Besides, there was an insignificant difference between the normal group and rats pretreated with TMB-NLC regarding macroscopic damage score and colon weight-to-length ratio.

#### Histopathological examination

##### H&E stain

**Figure 10.**
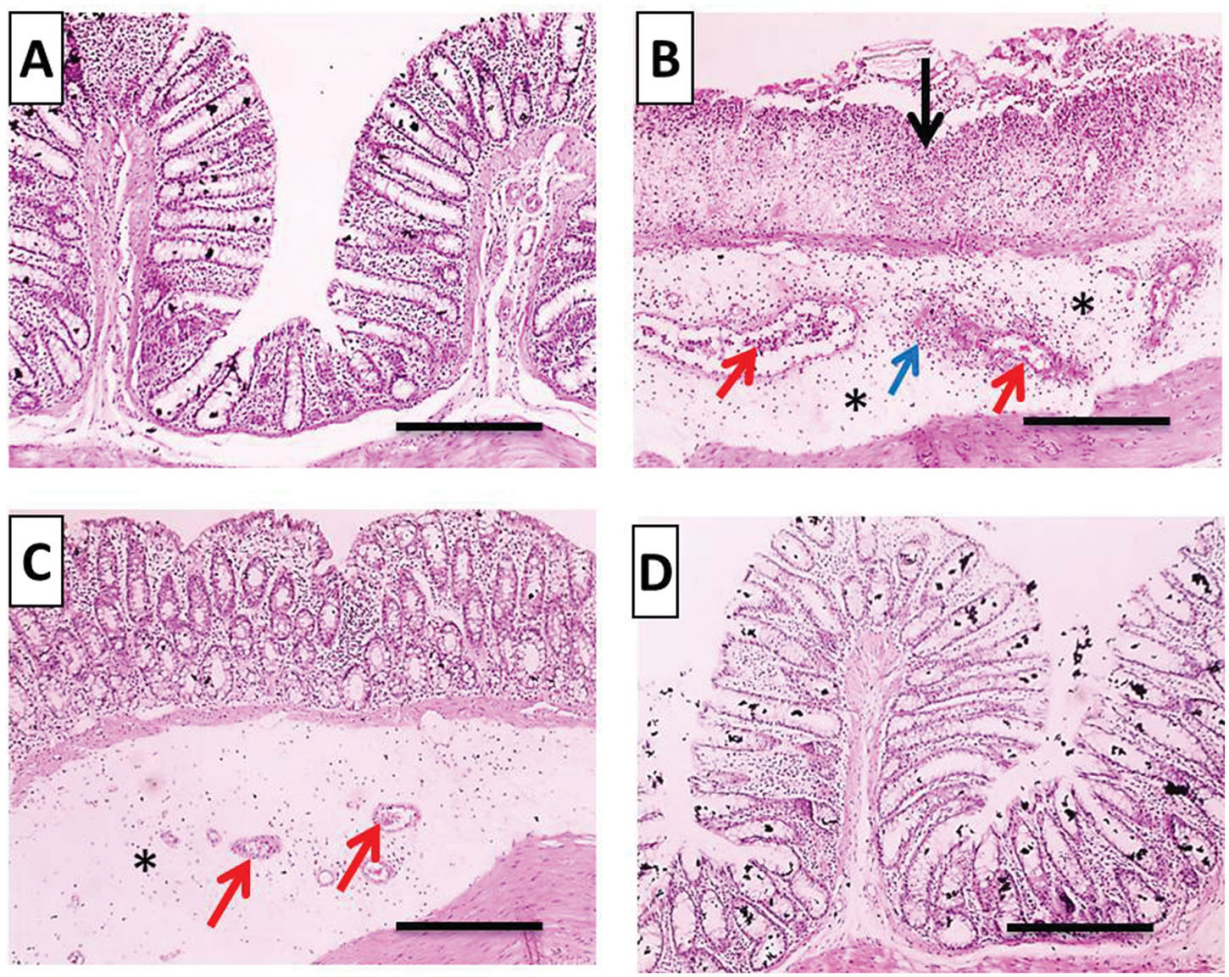
Histopathological examination (hematoxylin and eosin stained, ×100) of colonic tissues after acetic acid-induced colitis in rats. (A) Normal group; (B) positive control group; (C) TMB group; (D) TMB-NLC group. Mucosal necrosis and ulceration (black arrow), congestion (red arrows), inflammatory cells infiltration (blue arrow), and edema (black asterisk).

Figure 10 displays microphotographs of histopathological examination using H&E stain of colonic tissues of the different groups. For instance, normal histopathological structures with tall, regular, and closely related crypts cells were preserved in the normal group (Figure 10(A)). Meanwhile, acetic acid caused disease pathologies and severe tissue damage in the positive control group including loss of epithelial lining, extensive mucosal ulceration with the absence of crypts, inflammatory cells infiltration accompanied with remarkable submucosal edema and congestion (Figure 10(B)). Pretreatment with free TMB showed mild separated crypts, moderate submucosal edema and congestion (Figure 10(C)). TMB-NLC pretreated rats exhibited the most obvious protective impact against acetic acid-induced colonic microscopic damage with regular cell arrangements and normal appearance of mucosa and submucosa (Figure 10(D)).

**Figure 11.**
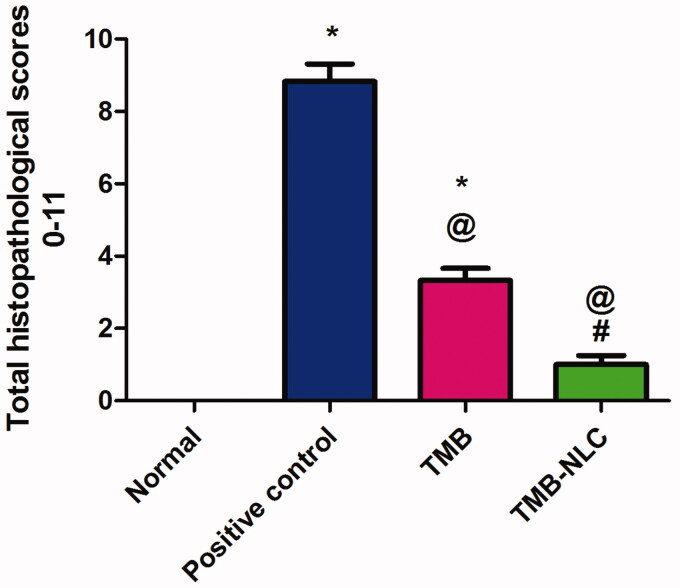
Statistical analysis of microscopic histopathological scores in colonic tissues. Data are mean ± SD, *n* = 6; statistical differences at *p*<.05 considered significant; *vs. normal group; ^@^vs. positive control group; ^#^vs. TMB group.

Microscopic scores of histopathological examination of colonic tissues were assigned based on the severity of colon injury on a scale of 0–11 (Figure 11). The beneficial effect of oral pretreatment with TMB either free or as NLC significantly lowered (*p*<.05) the histopathological scores in comparison with the positive control group. In contrast, oral administration of TMB loaded NLC resulted in significantly (*p*<.05) lower histopathological scores than rats given free TMB. Moreover, there was an insignificant difference between normal control and rats that received TMB loaded NLC.

##### Alcian blue stain

The mucus layer is widely known to enhance the repair of chemically induced epithelium damage as well as prevent diarrhea and blood loss through stool (Awaad et al., 2017).

**Figure 12.**
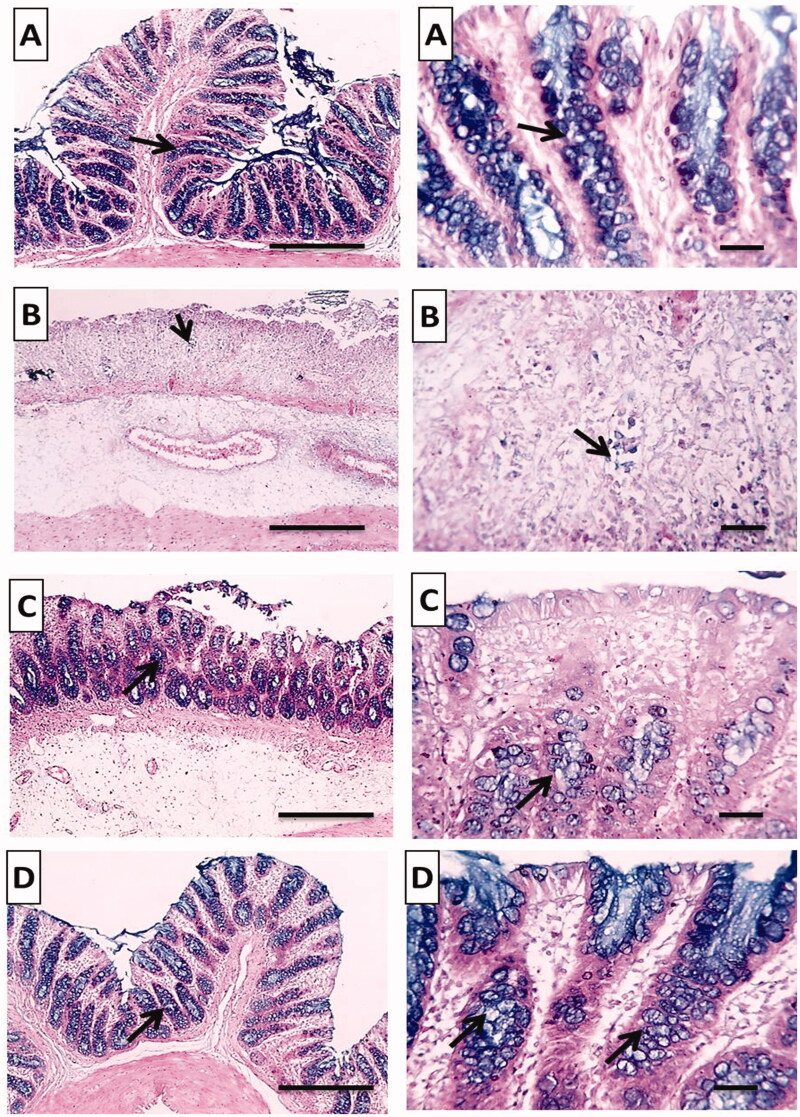
Histochemical examination (Alcian blue stain) of colonic tissues after acetic acid-induced colitis in rats to detect colonic mucus. (A) Normal group; (B) positive control group; (C) TMB group; (D) TMB-NLC group. Magnification, ×100/scale bar = 100 μm (left side) and ×400/scale bar = 50 μm (right side). Goblet cells lining crypts (black arrow).

The normal group stained strongly with Alcian blue and showed normal mucus secreting goblet cells lining the intestinal crypts (Figure 12(A)). On the other hand, there was a very weak reaction in the positive control group indicating glandular atrophy and severe loss of mucus secreting goblet cells (Figure 12(B)). Pretreated rat groups with free TMB showed moderate reaction and increase in the numbers of mucus secreting goblet cells stained with Alcian blue (Figure 12(C)). Most interestingly, TMB loaded NLC showed a strong diffuse cytoplasmic Alcian blue in the goblet cells lining indicating retained normal amount of mucus secreting goblet cells lining the intestinal crypts (Figure 12(D)).

According to our findings, treatment with TMB loaded NLC could decrease macroscopic and microscopic damage scores with normal colonic mucus. This could be attributed to the sustained release of TMB from NLC at the site of inflammation promoting TMB local anesthetic activity (Chevalier et al., 2004). Besides, anionic nano-delivery systems were selectively attached via electrostatic interaction to the positively charged cationic proteins which increased in the inflamed tissues (Hua et al., 2015). Moreover, capryol 90 improved the intestinal absorption of TMB owing to its absorption enhancing mechanisms via both transcellular and paracellular pathways (Ukai et al., 2020). The paracellular pathway or endocytosis, which is extensively explored for the delivery of nanocarriers directly through the colon inflamed tissues where the drug is released promoting local pharmacological effect (Nedelcu et al., 2021).

### Immunohistochemical localization of TNF*-*α

TNF*-*α is the orchestrating molecule of various pathogenic processes and plays essential role in UC to evaluate the severity of inflammation (Can et al., 2015; Owusu et al., 2020). TNF*-*α is a strong cytokine, influences the mucosal immune system, alters epithelial integrity, controls macrophage and neutrophil infiltration at the site of inflammation (Can et al., 2015). As well, it is involved in increased endothelial cell permeability, leukocyte production and activation to generate more PGs (Owusu et al., 2020).

**Figure 13.**
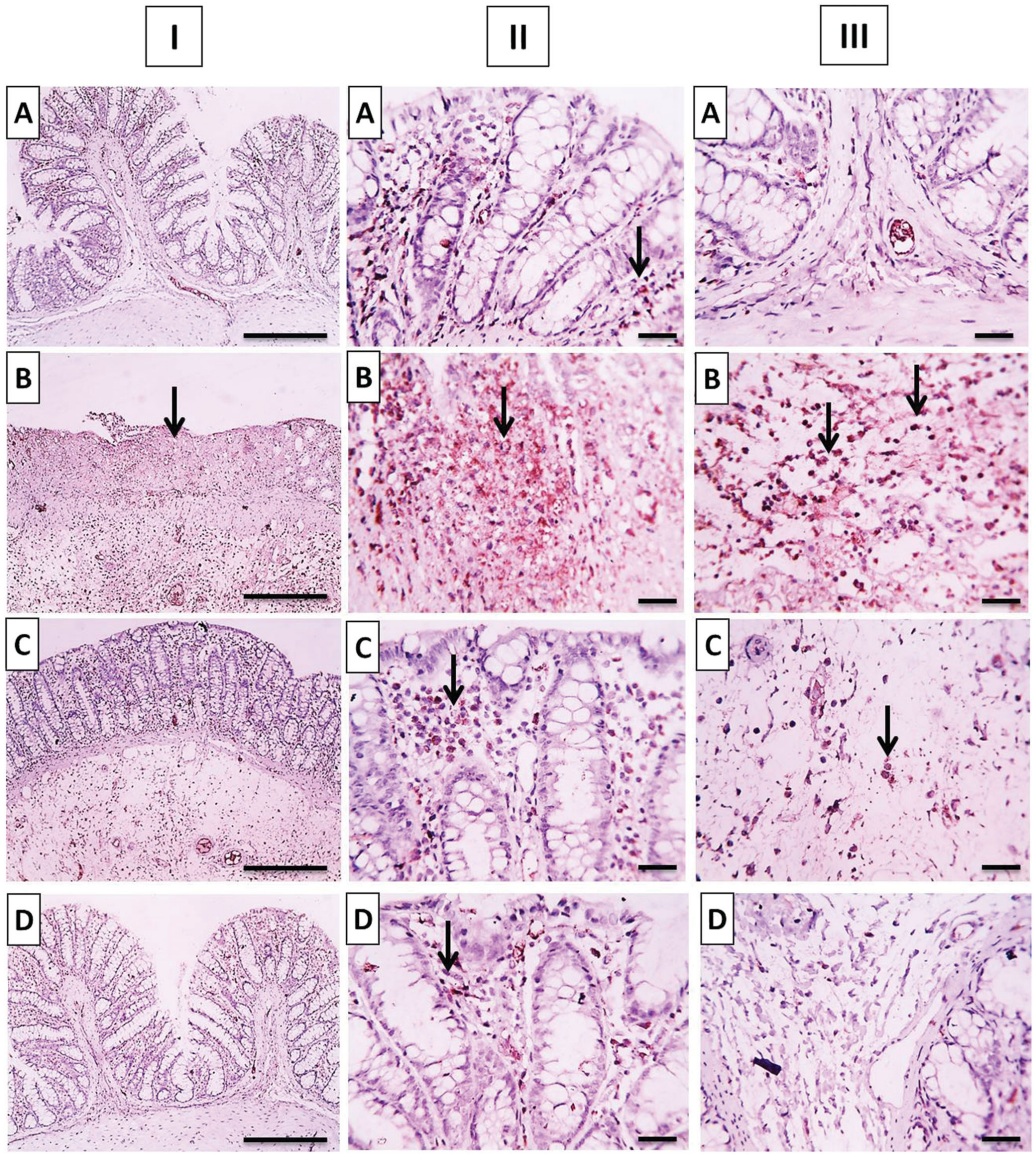
Photomicrographs of (I) whole colonic tissues, (II) mucosa, and (III) submucosa immunostained against TNF-α after acetic acid-induced colitis in rats. (A) Normal group; (B) positive control group; (C) TMB group; (D) TMB-NLC group. Magnification, ×100/scale bar = 100 μm (the whole section) and ×400/scale bar = 50 μm (the mucosal and submucosal parts). Positive stainings are shown by black arrows.

Positive immunohistochemical staining of TNF-α was recorded as a brown cytoplasmic reaction in the mucosal and submucosal colonic parts. The normal group showed minimal TNF*-*α expression in mucosa without reaction in the submucosa (Figure 13(A)). The positive control group showed positive dense TNF*-*α expression in the mucosa and in inflammatory cells infiltrating submucosa (Figure 13(B)). The TNF-α expression decreased in the free TMB pretreated group which was moderate in mucosa besides a few inflammatory cells infiltrating the submucosa (Figure 13(C)). Whereas, mild positive TNF-α expression was observed in the mucosa in rats pretreated with TMB loaded NLC with slight reaction in the submucosa (Figure 13(D)).

**Figure 14.**
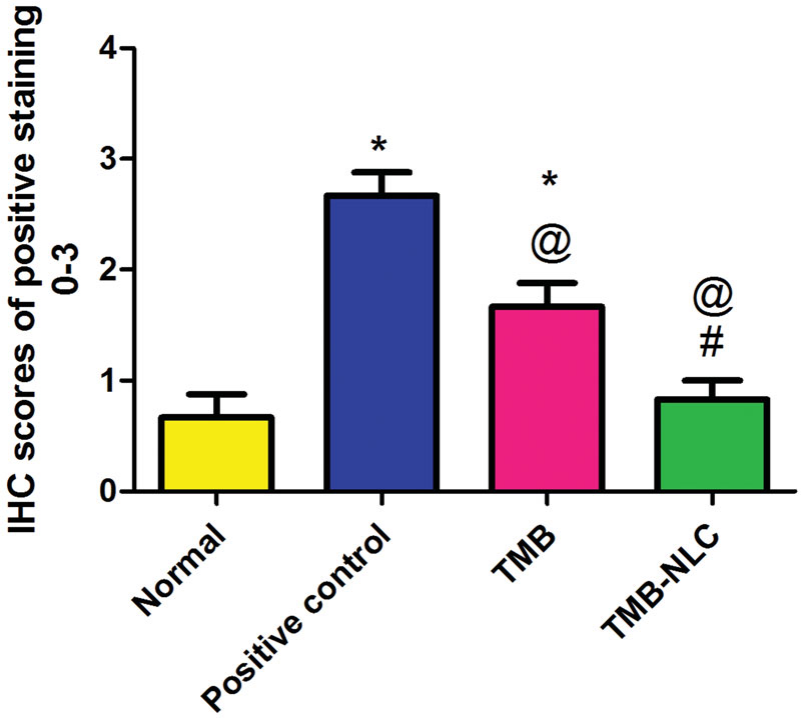
Statistical analysis of IHC staining intensity of TNF-α in colonic tissues. Data are mean ± SD, *n* = 6. Statistical differences at *p*<.05 were considered significant; *vs. normal group; ^@^vs. positive control group; ^#^vs. TMB group.

The result of statistical analysis of immunohistochemical localization of TNF*-*α in colonic tissue is shown in Figure 14. In positive control, there was a significant (*p*<.05) increase in TNF*-*α expression when compared to the normal group. Oral pretreatment with free TMB and TMB loaded NLC significantly reduced the immunoreactivity when compared to the positive control. The superiority of TMB-NLC could be inferred by their significantly (*p*<.05) reduction in TNF*-*α expression in comparison with free TMB as well as the insignificantly different TNF*-*α expression from that recorded in the normal group.

The dominance of the TMB-NLC over free TMB in decreasing the level of TNF*-*α localization could be attributed to the sustained release of TMB from NLC at the site of UC. This promotes the anti-inflammatory properties which can be related to TMB local anesthetic effect via blocking the sodium channels affecting TNF*-*α upregulation and inhibiting its role in damage and inflammation (Chevalier et al., 2004). Hence, TMB-NLC could effectively reduce inflammation, improve hypercoagulable state and promote the recovery of the disease (Gao et al., 2017).

### Assessment of oxidative stress markers

GSH is a significant component of the antioxidant defense of most tissues at relatively high intracellular concentrations promoting a cytoprotective action to cells. Moreover, depletion of tissue GSH associated with a marked cellular degeneration of the colon epithelium even in the absence of treatments to increase oxidative stress above the basal level (Holmes et al., 1998).

In healthy rats, SOD plays important role in cell protection against ulcerative injury by promoting superoxide anion dismutation and inhibiting lipid peroxidation as well as preventing leukocyte rolling and adhesion to colonic tissues (Baldo et al., 2016).

Malondialdehyde is a byproduct of lipid peroxidation occurring in the inflamed tissues indicating peroxide damage to the membrane (Anwer et al., 2020).

**Figure 15.**
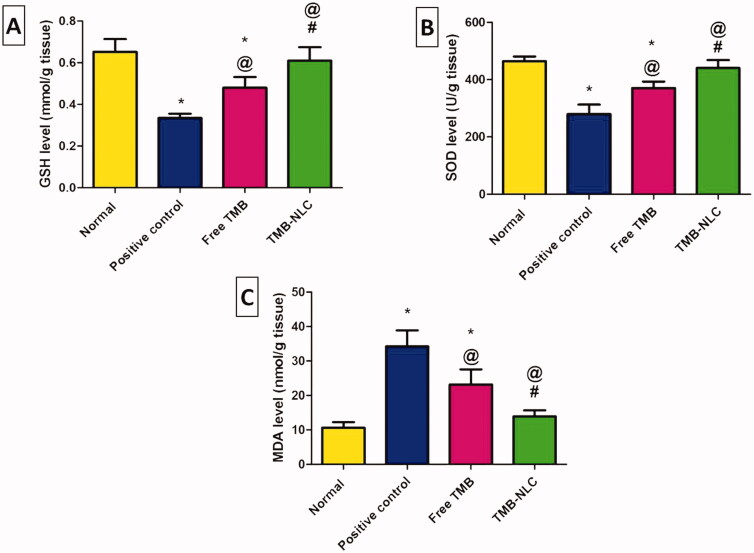
Effects of TMB oral pretreatment on acetic acid-induced ulcerative colitis in oxidative stress markers of (A) GSH, (B) SOD, and (C) MDA in the colonic tissue. Data are mean ± SD, *n* = 6. Statistical differences at *p*<.05 were considered significant; *vs. normal group; ^@^vs. positive control group; ^#^vs. TMB group.

The levels of the oxidative stress markers including GSH, SOD, and MDA in the tissue homogenates of all the experimental rats are shown in Figure 15. Intra-rectally administration of acetic acid (4%, 2 mL/rat) to the positive control group induced a significant decrease in GSH (Figure 15(A)) and SOD (Figure 15(B)) activities accompanied by a significant increase in MDA content (Figure 15(C)) when compared to the normal group. Oral pretreatment with free TMB and TMB loaded NLC significantly elevated the levels of GSH and SOD together with a significant lowering in MDA content in comparison with the positive control group. The significant elevation of GSH and SOD levels and the significant reduction of MDA content demonstrated also the superiority of the NLC over the free TMB as well as being insignificantly different from those recorded in the normal group (Figure 15(D)).

In agreement with previous *in vivo* studies (Hua et al., 2015), our findings have shown promising results for anionic nano-delivery systems in IBD. Thus, it can be said that NLC significantly potentiated the protective effect of TMB against acetic acid-induced colitis in rats.

## Conclusion

In the present study, a novel biocompatible nanoformulation for TMB has been developed with colon specific properties. The optimized NLC possessed favorable characteristics, showed sustained release of the drug in physiological buffer, and was stable over the course of 30 days, with no notable changes in the PDI, ZP, or particle size. The total disappearance of the TMB signals in the solid-state characterization spectra conveyed an optimal drug encapsulation within the matrix in an amorphous state and good compatibility between the drug and NLC components. The optimized NLC (F9) exhibited a protective activity against acetic acid-induced UC in rats via maintaining colonic tissues architecture, histopathological alterations, TNF*-*α expression, and the colonic tissue levels of the oxidative stress markers. Therefore, this study may highlight the NLC as a potential oral drug delivery system for TMB to alleviate the severity of UC and encourage prospective clinical investigation. Based on large-scale prospective studies, future work is warranted to interpret in-depth the aforesaid anticipated TMB promising effects on GIT disorders.
